# The association of FAT1 mutations with therapeutic outcomes in AML, especially in receiving venetoclax combination

**DOI:** 10.3389/fonc.2025.1456832

**Published:** 2025-03-31

**Authors:** Tao Wang, Junxia Huang, Yongqian Jia, Junting Li, Weiwei Mou, Guang Lu

**Affiliations:** ^1^ Department of Hematology, West China Hospital, Sichuan University, Chengdu, Sichuan, China; ^2^ Department of Hematology, Affiliated Hospital of Qingdao University, Qiangdao, China; ^3^ Department of Clinical Medicine, Shanghai Jiao Tong University School of Medicine, Shanghai, China; ^4^ Department of Hematology, Shengli Oilfield Central Hospital, Dongying, Shandong, China; ^5^ Department of Hematology, Shandong Second Provincial General Hospital, Jinan, Shandong, China

**Keywords:** AML, FAT1, venetoclax (ABT-199), mutation, therapeutic outcome

## Abstract

**Objective:**

The role of FAT1 mutations in acute myeloid leukemia (AML) remains unclear, particularly regarding their impact on the chemosensitivity of AML patients. To elucidate the effect of FAT1 mutations on the therapeutic outcomes and prognosis of AML patients, we conducted this study.

**Methods:**

To analyze the impact of FAT1 mutations, we obtained data from the LAML-KR cohort of the International Cancer Genome Consortium (ICGC), consisting of 205 patients. Additionally, we retrospectively collected data from 108 primary AML patients who received initial induction chemotherapy with a venetoclax combination regimen between January 2019 and December 2023 at Qingdao Medical College Affiliated Hospital and Shengli Oilfield Central Hospital (referred to as the Venetoclax-AML cohort). We analyzed the characteristics, clinical features, and molecular genetic features of FAT1 mutations, and assessed the impact of FAT1 mutations on therapeutic outcomes and prognosis in both cohorts.

**Results:**

In the public LAML-KR cohort (n = 205), the mutation rate of the FAT1 gene was approximately 15% (31/205), with a nonsynonymous mutation rate of about 6%. Patients with FAT1 mutations (including synonymous mutations) exhibited a higher tumor mutation burden (TMB) compared to wild-type patients (p < 0.001). Further analysis of the 83 patients in the LAML-KR cohort with complete clinical data showed that the mutation rates of P53, DNMT3A, FLT3, and NPM1 genes (including synonymous mutations) were higher in the FAT1 mutant group than in the wild-type group (p < 0.05). In our retrospective Venetoclax-AML cohort (n = 108), the nonsynonymous mutation rate of the FAT1 gene was approximately 13% (14/108), which was higher than the mutation rate in the public LAML-KR cohort. Moreover, only the P53 mutation rate was higher in FAT1 mutant patients (p < 0.01), while the mutation rates of DNMT3A, FLT3, and NPM1 genes showed no significant difference between FAT1 mutant and wild-type patients (p > 0.05). In both the LAML-KR and Venetoclax-AML cohorts, FAT1 mutant patients showed better initial induction chemotherapy outcomes compared to wild-type patients. However, in the LAML-KR cohort, there was no improvement in overall survival (OS) for FAT1 mutant patients (median survival time: 34.6 months vs. 41.7 months, p = 0.6757), whereas there was a trend toward improved progression-free survival (PFS) in the Venetoclax-AML cohort (p = 0.103).Interestingly, further analysis of P53 mutant patients (n = 17) in the Venetoclax-AML cohort revealed that FAT1 mutant patients had better initial induction chemotherapy outcomes and a trend toward improved PFS compared to wild-type patients (p = 0.1381).

**Conclusion:**

AML patients with FAT1 mutations have better initial induction chemotherapy efficacy with venetoclax-based regimens compared to wild-type patients, and there is a trend toward improved PFS. This may be related to the improved efficacy and prognosis in P53 mutation-positive patients.

## 1 Introduction

Acute myeloid leukemia (AML) is the most common type of acute leukemia in adults. It progresses rapidly, is highly aggressive, and remains a challenging disease to treat (1). AML can affect individuals of all ages, with approximately two-thirds of patients being over 55 years old, and the median age at diagnosis being 68 years, making it most common in the elderly (2). Globally, over 80,000 AML-related deaths occur annually, and with the increasing global aging population, the incidence is expected to double over the next 20 years (3), posing a significant threat to human health. In the past five years, there have been substantial changes in the treatment regimens for newly diagnosed AML patients, largely due to the increased availability of small molecule targeted drugs, such as venetoclax (4–6). This has significantly improved the prognosis of AML patients, with the 5-year relative survival rate currently rising to 31.9% (7). However, AML continues to face numerous challenges and difficulties.

Venetoclax, formerly known as ABT-199, is a BH3 mimetic and a selective, potent inhibitor of the BCL2 protein. Based primarily on the results of the Phase III studies VIALE-A (NCT02993523) and VIALE-C (NCT03069352) (8, 9), the U.S. Food and Drug Administration (FDA) and the European Medicines Agency (EMA) have approved venetoclax in combination with hypomethylating agents (HMAs) or low-dose cytarabine (LDAC) for the treatment of newly diagnosed AML in patients aged ≥75 years or those who are ineligible for intensive chemotherapy. The high response rates and improved survival observed in these patients have led many medical centers to consider venetoclax combination regimens for elderly patients without significant comorbidities and even for younger patients with high-risk disease features, due to the poor expected outcomes of intensive chemotherapy in these groups. Furthermore, venetoclax combined with HMAs has also shown efficacy in relapsed AML patients, particularly those who relapse after intensive chemotherapy (10, 11). This regimen also can significantly enhance the feasibility of allogeneic hematopoietic stem cell transplantation (allo-HSCT) in older patients deemed fit for transplantation (12). With the widespread application of venetoclax-based regimens in AML, it is crucial to identify factors that can predict or influence the efficacy of venetoclax and to overcome the resistance that may arise from its long-term use (13).

Human atypical cadherin FAT1 was cloned from a human T-leukemia cell line in 1995 and is located on chromosome 4q34-35, consisting of 27 exons. FAT1, acting as a molecular “brake” for mitochondrial respiration, regulates the proliferation and migration of vascular smooth muscle cells during vascular injury (14, 15). It also serves as a receptor in signaling pathways that regulate cell-cell contact interactions and planar cell polarity (16, 17). Additionally, FAT1 has been shown to be closely related to human diseases, particularly as one of the most frequently mutated genes in human tumors. Studies have indicated that FAT1 can influence tumor cell proliferation, migration, invasion, stemness, and epithelial-mesenchymal transition (EMT) by regulating multiple signaling pathways, including Wnt/β-catenin, Hippo, and MAPK/ERK (18, 19). FAT1 is also significantly correlated with exosome markers, immune cell markers, methylation marker genes, hypoxia-related mutated genes, and autophagy-related marker genes in various tumors (20). Research suggests that FAT1 may play a role in either blocking or promoting carcinogenesis and cancer progression depending on the type of cancer. However, studies on the biological role of FAT1 in AML are currently scarce, and its function in AML remains unclear. In a study by Garg et al., whole-exome sequencing was performed on 13 matched samples at diagnosis, relapse, and remission, followed by targeted sequencing of 299 genes in 67 AML patients with FLT3-ITD mutations, identifying an unexpectedly high FAT1 mutation rate of up to 10% (8/80). Recently, researchers have found that FAT1 can reduce autophagy levels and proliferation activity in AML cells by downregulating ATG4B expression through the inhibition of TGFβ-Smad2/3 signaling, suggesting that FAT1 might be a new target for developing AML-targeted therapies (21).

In summary, FAT1 plays a significant role in the development, progression, and drug resistance of tumors, but its role in AML remains unclear. Given that FAT1 is one of the most frequently mutated genes in human tumors, we hypothesized that FAT1 mutations in AML might affect the therapeutic outcomes and prognosis of AML patients. Therefore, in this study, we analyzed the characteristics, clinical features, and molecular genetic features of FAT1 mutations in AML and investigated the impact of FAT1 mutations on the therapeutic outcomes and prognosis of AML patients, particularly those receiving venetoclax combination therapy.

## 2 Methods

### 2.1 Source of FAT1 gene mutation data

The public data for FAT1 mutation analysis were obtained from the LAML-KR cohort of the International Cancer Genome Consortium (ICGC), which includes information on 205 AML patients. Mutation data for all patients were available, and all had received chemotherapy, although the specific chemotherapy regimens were not disclosed. Clinical data were complete for 83 of these patients. Additionally, we retrospectively collected data from 108 primary AML patients who received initial induction chemotherapy with a venetoclax combination regimen between January 2019 and December 2023 at Qingdao Medical College Affiliated Hospital and Shengli Oilfield Central Hospital, referred to as the Venetoclax-AML cohort. The venetoclax combination regimens included: venetoclax + azacitidine, venetoclax + decitabine, venetoclax + intensive chemotherapy (IC), and venetoclax + other agents (OA), with each patient receiving at least two treatment cycles. The Venetoclax-AML cohort included 108 patients for genetic mutation and therapeutic efficacy analysis, and 94 patients were ultimately included in the progression-free survival (PFS) analysis based on the completeness of survival data. The data collection for the Venetoclax-AML cohort was approved by the ethics committee, with the ethics number: YXLL202410201.

### 2.2 Collection and analysis of clinical data related to FAT1 mutations

Inclusion Criteria: (1):Age ≥ 14 years;(2)Diagnosed with AML. Exclusion Criteria: Incomplete data. The main clinical information collected included age, gender, survival time, survival status, risk stratification, efficacy, and molecular genetics. The diagnostic criteria for AML, efficacy evaluation, and AML risk stratification were based on the “Chinese Guidelines for the Diagnosis and Treatment of Adult Acute Myeloid Leukemia (Non-Acute Promyelocytic Leukemia) (2023 Edition)” and the 2022 European Leukemia Net (ELN) guidelines for diagnosis and treatment (22, 23).

The follow-up period for patients started from the first day of hospitalization, with the cut-off date being January 2023. Follow-up methods included outpatient and inpatient medical records and telephone follow-ups. Progression-free survival (PFS) was defined as the time from the start of treatment to disease progression or patient death (whichever occurred first). Overall survival (OS) was defined as the time from the start of treatment to patient death. Overall response rate (ORR) was defined as the sum of the proportions of patients achieving complete remission (CR), complete remission with incomplete hematologic recovery (CRi), or partial remission (PR). Non-complete remission (Non-CR) was defined as the sum of the proportions of patients with partial remission (PR) or non-remission (NR).

### 2.3 Analysis of FAT1 mutation sequencing data

The somatic single nucleotide mutation (SSM) data of patients in the LAML-KR cohort were analyzed using the R package ‘maftools’ (24). Tumor mutation burden (TMB) was defined as the number of accumulated mutations per megabase (Mb) in the coding region of tumor cells. Patients in the LAML-KR cohort were divided into TMB-high and TMB-low groups based on the median TMB value. For the Venetoclax-AML cohort, mutation data were detected and provided by Tianjin Xiehe Jingbo Medical Diagnostic Technology Co., Ltd., and Tianjin Jiankang Huamei Medical Testing Laboratory. The samples used for detection were bone marrow cells, analyzed using the Illumina high-throughput sequencing platform, with GRCh37/hg19 as the reference genome.

### 2.4 Statistical analysis of FAT1 mutation data

Statistical analyses and tests were performed using R software (version 4.2.2; The R Foundation for Statistical Computing, Vienna, Austria). Various categorical variables were analyzed using the chi-square test or Fisher’s exact test to calculate p-values. Kaplan-Meier (KM) and log-rank tests were used to analyze relevant clinical survival data. All tests were considered statistically significant at P < 0.05.

## 3 Results

### 3.1 Mutation frequency and characteristics of FAT1 in the LAML-KR cohort

**Figure 1:**
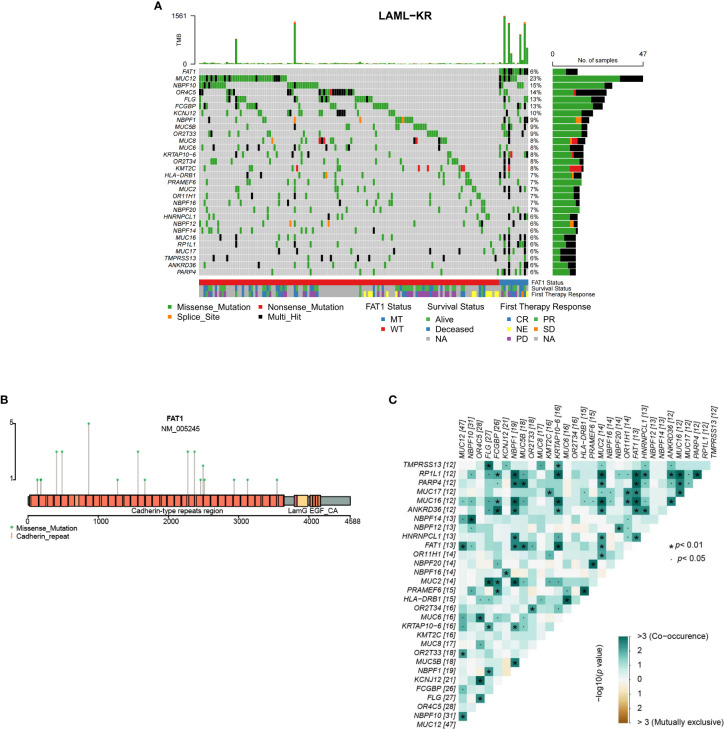
Overview of FAT1 mutations in the LAML-KR cohort. **(A)** The oncoplot shows the top 30 genes with the highest mutation rates in the LAML-KR cohort. FAT1 has a high mutation frequency in this cohort, and patients with FAT1 mutations have a higher tumor mutation burden (TMB); **(B)** The mutation sites of FAT1 in the LAML-KR cohort are mainly concentrated in the cadherin repeat regions; **(C)** The heatmap shows the co-occurrence and mutual exclusivity of the top 30 mutated genes in the LAML-KR cohort. *: represents statistically significant relationships between gene pairs.

FAT1 is one of the most frequently mutated genes in human tumors, with oncogene or tumor suppressor gene mutations often leading to dysregulated gene expression, such as P53. To study the impact of FAT1 mutations on the therapeutic outcomes and prognosis of AML patients, we first performed bioinformatics analysis of the FAT1 mutation spectrum in 205 patients from the ICGC public database LAML-KR cohort. **Figure 1A** shows the top 30 genes with the highest mutation rates in the LAML-KR cohort, where the FAT1 gene mutation rate is approximately 15% (31/205), with a nonsynonymous mutation rate of about 6% (13/205), indicating a relatively high mutation rate in this cohort (**Figure 1A**). Among the nonsynonymous mutation types of the FAT1 gene, almost all are missense mutations, primarily concentrated in the cadherin repeat regions (**Figure 1B**). Notably, patients with FAT1 mutations have a higher tumor mutation burden (TMB) compared to wild-type patients (p < 0.001, **Figure 1A**, **Table 1**). Furthermore, we analyzed the co-occurrence and mutual exclusivity of the top 30 genes with the highest mutation frequencies in the LAML-KR cohort (**Figure 1C**). Among them, the KMT2C gene, a member of the Mixed Lineage Leukemia (MLL) family, also had a high mutation rate in this cohort, and MLL gene rearrangements are common genetic alterations in AML.

**Table 1:** Comparison of clinical and molecular genetic characteristics between FAT1 wild-type (WT) and FAT1 mutant (MT) patients in the LAML-KR cohort.

	FAT1-WT	FAT1-MT	P.value
	*N=62*	*N=21*	
Age, years, n (%)			1.000
<=65	52 (62.65)	17 (20.48)	
>65	10 (12.05)	4 (4.82)	
Sex, n (%)			0.654
male	35 (42.17)	10 (12.05)	
female	27 (32.53)	11 (13.25)	
Survival Status, n (%)			0.267
Alive	40 (48.19)	10 (12.05)	
Deceased	22 (26.51)	11 (13.25)	
First Therapy Response, n (%)			0.009
CR	20 (24.1)	14 (16.9)	
PR	8 (9.6)	2 (2.4)	
SD	0 (0.00)	1 (1.2)	
PD	33 (39.8)	4 (4.8)	
NE	1 (1.2)	0 (0.00)	
TMB*, n (%)			<0.001
High	75 (36.59)	26 (12.68)	
Low	99 (48.29)	5 (2.44)	
TP53 mutation*, n (%)	1 (0.5)	6 (2.9)	<0.0001
DNMT3A mutation*, n (%)	14 (6.83)	10 (4.88)	0.0007
FLT3 mutation*, n (%)	13 (6.34)	7 (3.41)	0.01
NPM1 mutation*, n (%)	4 (1.95)	5 (2.44)	0.047

*Mutation-related data for all 205 patients in the LAML-KR cohort were available (FAT1-WT: n=174, FAT1-MT: n=31).

*All gene mutation types include synonymous mutations.

### 3.2 Clinical and molecular genetic characteristics of FAT1 mutant patients in AML

To investigate the clinical significance of FAT1 mutations in AML, we analyzed the clinical and molecular genetic characteristics of FAT1 mutant patients in both the LAML-KR cohort and the Venetoclax-AML cohort. In the LAML-KR cohort, limited by the completeness of patient data, we included a total of 83 patients with complete clinical data for analysis. The results showed that there were no significant differences between FAT1 mutant (including synonymous mutations) patients (n=21) and wild-type patients (n=62) in terms of age, gender, and survival status (p > 0.05, **Table 1**). Additionally, the mutation rates of P53, DNMT3A, FLT3, and NPM1 genes were significantly higher in FAT1 mutant patients (including synonymous mutations) compared to FAT1 wild-type patients, with the differences being statistically significant (p < 0.05, **Table 1**).

**Figure 2:**
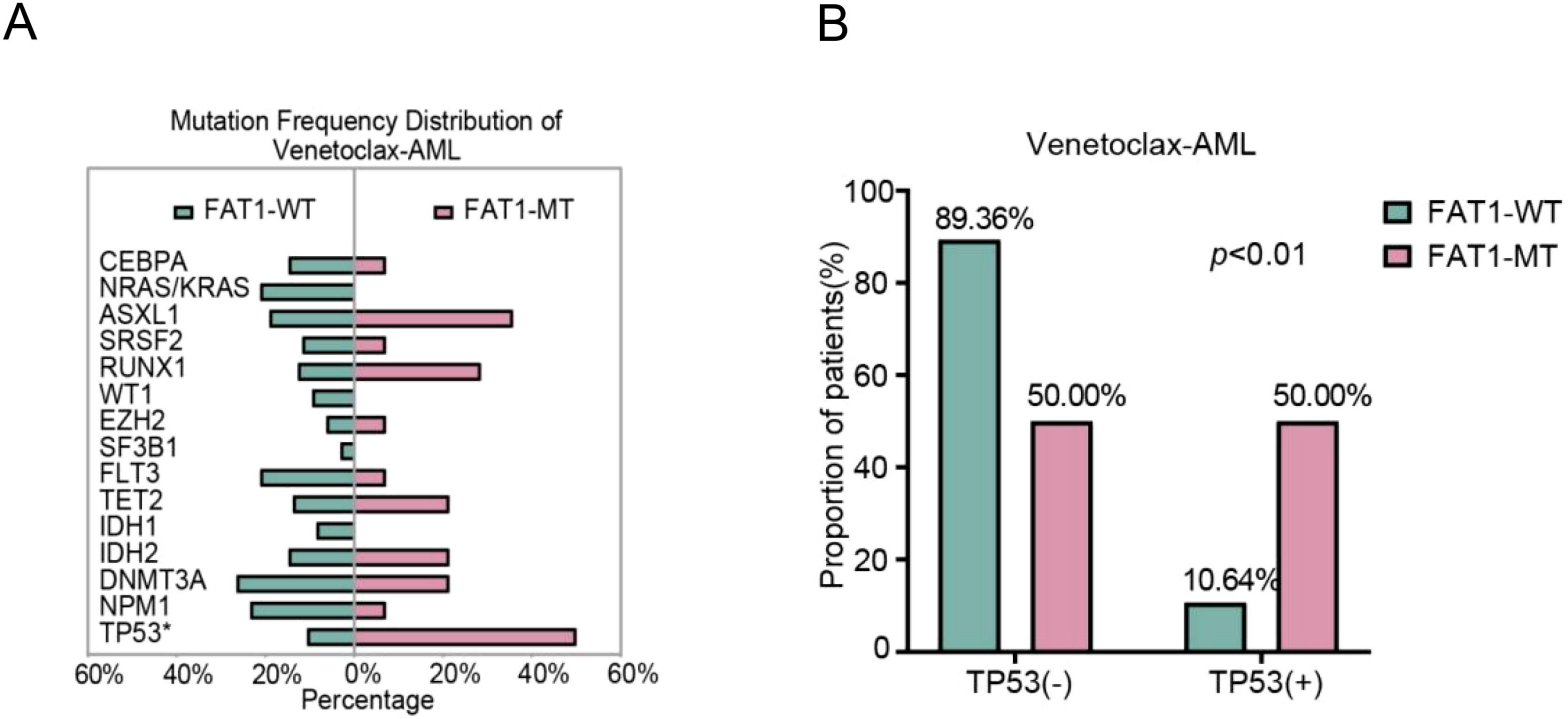
Distribution of AML-related mutant genes in the venetoclax-AML cohort. **(A)** Distribution of AML-related gene mutation rates between FAT1 wild-type (WT) and FAT1 mutant (MT) patients, with a significantly higher P53 mutation rate in FAT1 mutant (MT) patients (p < 0.01); **(B)** Comparison of TP53 gene mutation rates between FAT1 wild-type (WT) and FAT1 mutant (MT) patients in the Venetoclax-AML cohort.

In the Venetoclax-AML cohort, the nonsynonymous mutation rate of the FAT1 gene was approximately 13% (14/108). Compared to wild-type patients (n=94), FAT1 mutant patients (n=14) showed no significant differences in age, gender, survival status, performance status score, AML type (primary or secondary), ELN2022 risk stratification, and treatment regimen type (p > 0.05, **Table 2**). To verify the differences in P53, DNMT3A, FLT3, and NPM1 mutation rates under different FAT1 genetic statuses observed in the LAML-KR cohort, we conducted the same comparative analysis in the Venetoclax-AML cohort. The results showed that only the P53 mutation rate was significantly higher in FAT1 mutant patients compared to FAT1 wild-type patients (50.00% vs. 10.64%, p < 0.01, **Figure 2**). However, the mutation rates of DNMT3A, FLT3, and NPM1 genes showed no significant differences between FAT1 mutant and FAT1 wild-type patients (p > 0.05, **Table 2**, **Figure 2A**). These findings suggest a possible correlation between FAT1 mutations and P53 mutations.

**Table 2:** Comparison of clinical and molecular genetic characteristics between FAT1 wild-type (WT) and FAT1 mutant (MT) patients in the venetoclax-AML cohort.

	FAT1-WT	FAT1-MT	p.value
	*N=94*	*N=14*	
Age, years, n (%)			0.572
<=65	51 (47.22)	9 (8.33)	
>65	43 (39.82)	5 (4.63)	
Sex, n (%)			1.000
male	59 (54.63)	9 (8.33)	
female	35 (34.41)	5 (4.63)	
Survival Status, n (%)			0.286
Alive	62 (57.40)	12 (11.11)	
Deceased	21 (19.44)	1 (0.93)	
Missing	11 (10.19)	1 (0.93)	
Performance status, n (%)			0.770
≥2	32 (29.63)	4 (3.70)	
0-1	62 (57.41)	10 (9.26)	
Disease type, n (%)			1.000
*De novo*	78 (72.22)	12 (11.11)	
Secondary	16 (14.81)	2 (1.85)	
ELN2022, n (%)			0.223
Favorable	24 (22.22)	1 (0.93)	
Intermediate	23 (21.30)	1 (0.93)	
Adverse	45 (41.67)	12 (11.11)	
Failed	2 (1.85)	0 (0.00)	
Best Overall Response, n (%)			0.199
CR	54 (50.00)	12 (11.11)	
CR (MRD-)	34 (62.96)	10 (83.33)	
CR (MRD+)	20 (37.04)	2 (16.67)	
PR	11 (10.19)	2 (1.85)	
NR	22 (20.37)	0 (0.00)	
NE	7 (6.48)	0 (0.00)	
Therapeutic regimen, n (%)			0.238
Ven+Aza	59 (54.63)	10 (9.26)	
Ven+Dec	13 (12.03)	1 (0.93)	
Ven+IC	14 (12.96)	2 (1.85)	
Ven+OA	8 (7.401)	1 (0.93)	

### 3.3 Impact of FAT1 mutations on venetoclax-based treatment efficacy and prognosis in AML

**Figure 3:**
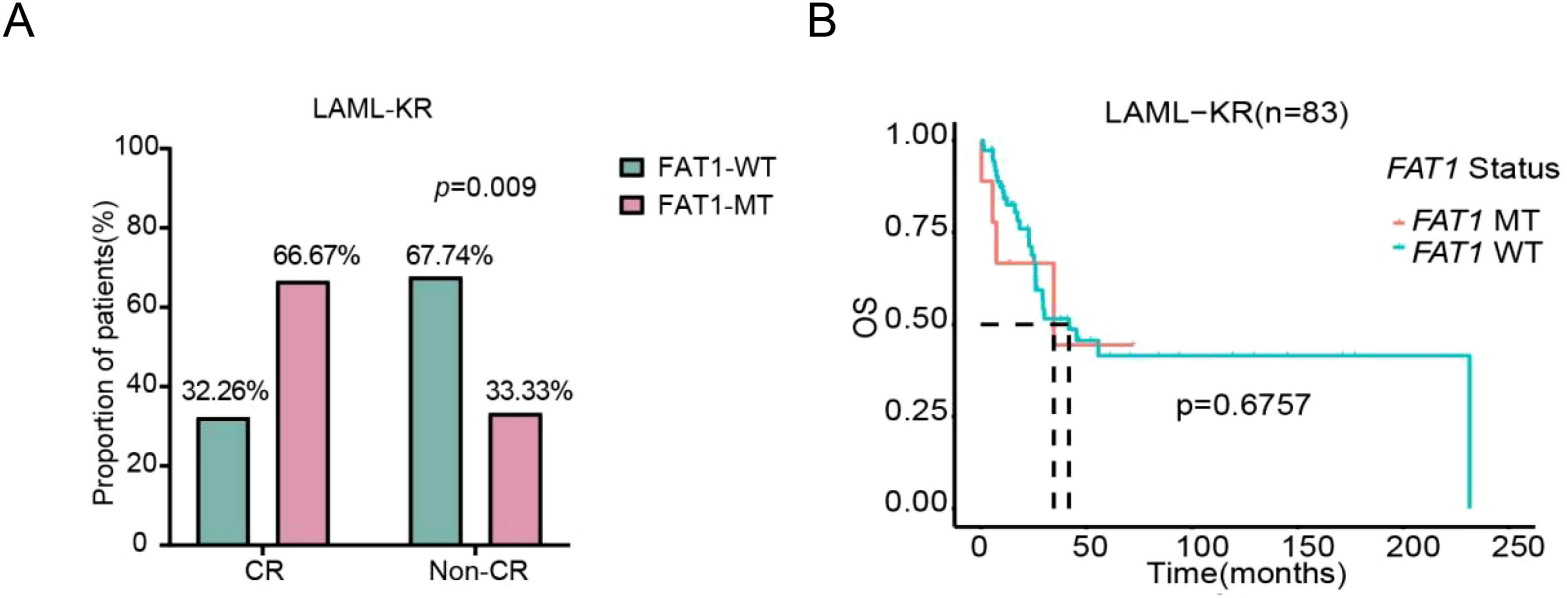
Comparison of initial induction chemotherapy response and prognosis between FAT1 wild-type (WT) and FAT1 mutant (MT) patients in the LAML-KR cohort. **(A)** FAT1 mutant patients achieved a higher CR rate compared to FAT1 wild-type patients (66.67% vs. 32.26%, p < 0.01); **(B)** Kaplan-Meier survival analysis of OS in FAT1 mutant and FAT1 wild-type patients (median survival time: 34.6 months vs. 41.7 months, p = 0.6757).

We further assessed the impact of FAT1 mutations on chemotherapy efficacy and prognosis in the LAML-KR cohort and the Venetoclax-AML cohort. In the LAML-KR cohort, FAT1 mutant patients (n=21) had a higher complete remission (CR) rate after initial induction chemotherapy compared to wild-type patients (n=62) (66.67% vs. 32.26%, p<0.01, **Figure 3A**). However, there was no significant survival advantage in overall survival (OS) for FAT1 mutant patients compared to wild-type patients (median survival time: 34.56 months vs. 41.67 months, p=0.6757, **Figure 3B**).

**Figure 4:**
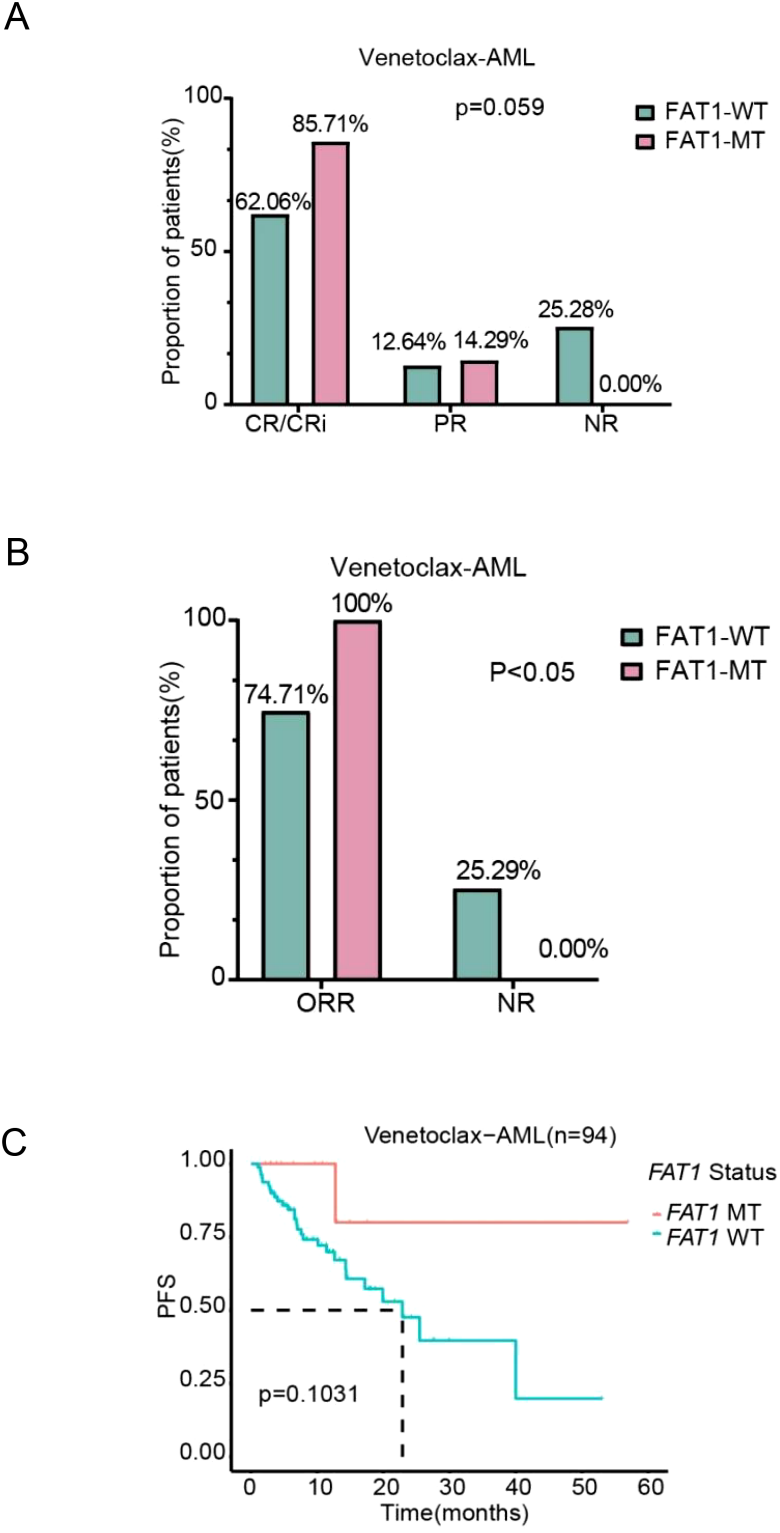
Comparison of chemotherapy efficacy and prognosis between FAT1 wild-type (WT) and FAT1 mutant (MT) patients in the LAML-KR cohort. **(A)** Efficacy stratification using CR/CRi, PR, and NR as indicators; **(B)** Efficacy stratification using ORR (CR/CRi + PR) and NR as indicators, showing that the ORR in FAT1 mutant patients was higher than in FAT1 wild-type patients (100% vs. 74.71%, p < 0.05); **(C)** Kaplan-Meier survival analysis of progression-free survival (PFS) comparing FAT1 wild-type (WT) and FAT1 mutant (MT) patients.

In the Venetoclax-AML cohort, when using CR/CRi, PR, and NR as efficacy classifications, there was no statistically significant difference between FAT1 mutant patients and wild-type patients in the CR/CRi rate (85.71% vs. 62.06%) and PR rate (14.29% vs. 12.64%) after initial induction chemotherapy (p > 0.05, **Figure 4A**). However, when using ORR (CR/CRi + PR) and NR as efficacy classifications, FAT1 mutant patients (n=14) achieved better therapeutic outcomes compared to wild-type patients (n=94) after initial induction chemotherapy (ORR: 100% vs. 74.71%, p < 0.05, **Figure 4B**). It is important to note that the venetoclax combination regimen is the first-line standard chemotherapy regimen for AML patients intolerant to intensive chemotherapy, making the use of ORR and NR as efficacy analysis parameters more reasonable. Due to the short follow-up period, OS data for most patients have not been obtained, and the number of FAT1 mutant cases is relatively small, so OS comparison analysis was not performed. Based on the completeness of survival data, we included 94 patients for PFS comparison analysis. The results showed that FAT1 mutant patients (n=13) exhibited a trend toward improved PFS compared to wild-type patients (n=81), although the difference was not statistically significant (p=0.1031, **Figure 4C**).

In summary, these results suggest that FAT1 mutations in AML are associated with better initial induction chemotherapy outcomes and continue to show superior efficacy in patients receiving venetoclax combination therapy. Although limited by the number of cases and follow-up duration, FAT1 mutations may contribute to improved survival benefits for patients.

### 3.4 Impact of FAT1 mutations on the efficacy and prognosis of venetoclax-based therapy in P53 mutant AML patients

**Figure 5:**
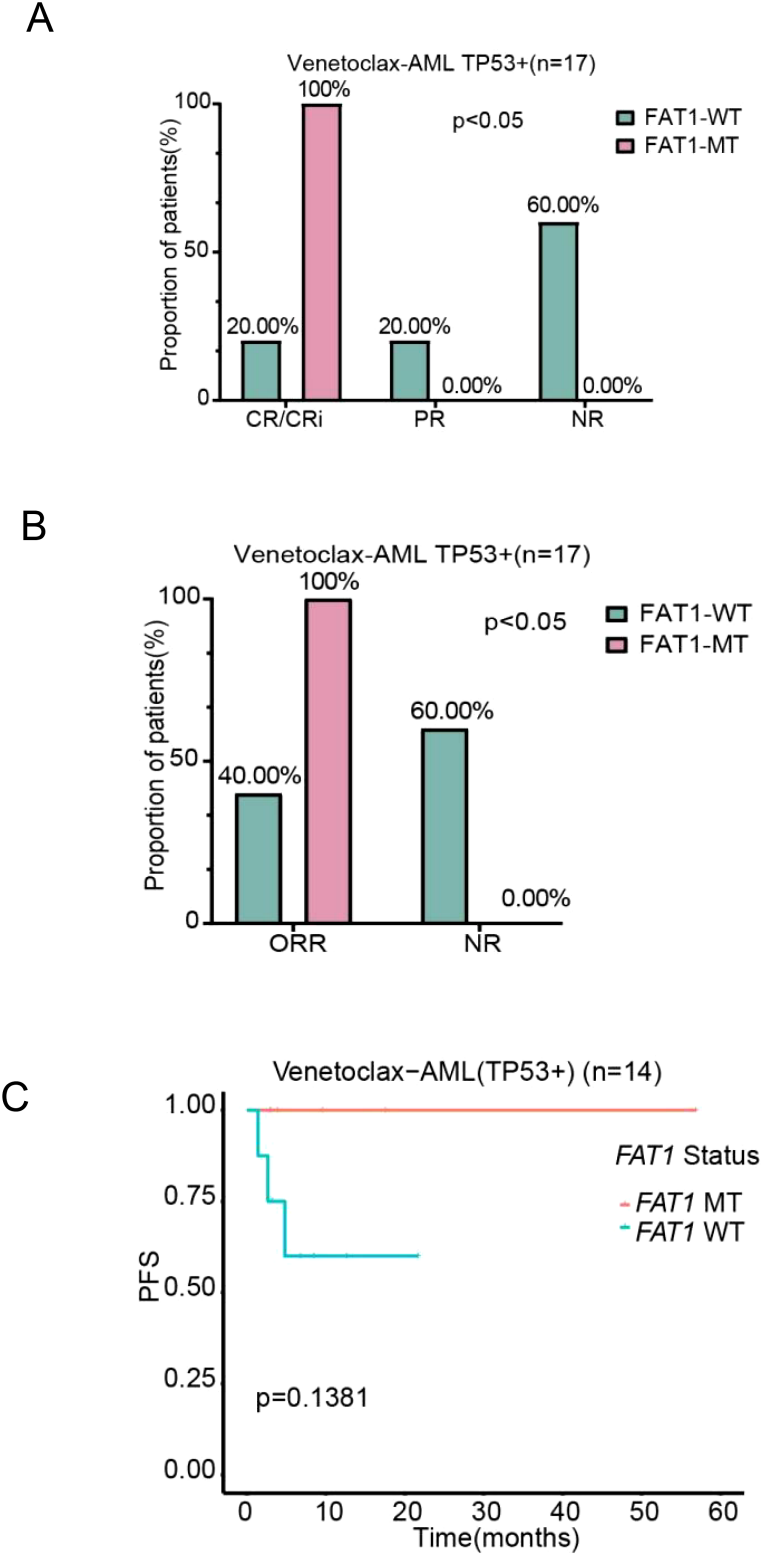
Comparison of chemotherapy efficacy and prognosis between FAT1 wild-type (WT) and FAT1 mutant (MT) patients in the P53 mutant positive subgroup of the venetoclax-AML cohort. **(A)** Efficacy stratification using CR/CRi, PR, and NR as indicators; **(B)** Efficacy stratification using ORR and NR as indicators; **(C)** Kaplan-Meier survival analysis of PFS in the P53 mutant positive subgroup, comparing FAT1 wild-type (WT) and FAT1 mutant (MT) patients.

Analysis of the molecular genetic characteristics of FAT1 mutant patients in the LAML-KR and Venetoclax-AML cohorts revealed a possible correlation between FAT1 and P53 mutations. Therefore, we further analyzed the impact of FAT1 mutations on AML patients carrying P53 mutations. Due to the small number of P53 mutant patients in the LAML-KR cohort (only 7 cases), we conducted the analysis only on P53 mutant patients in the Venetoclax-AML cohort. Based on the completeness of clinical data, a total of 17 P53 mutant patients in the Venetoclax-AML cohort were included for efficacy analysis, with 14 patients included in the PFS analysis. Regarding the efficacy of initial induction chemotherapy, whether using CR/CRi, PR, and NR as efficacy classifications or using ORR (CR/CRi + PR) and NR as efficacy classifications, the results showed that FAT1 mutant patients were associated with better therapeutic outcomes from the venetoclax combination regimen compared to wild-type patients (CR/CRi 100% vs. 20%, p = 0.002; ORR 100% vs. 40%, p = 0.03; **Figures 5A, B**). Additionally, there was a trend toward improved PFS (p = 0.1381; **Figure 5C**). These findings suggest that FAT1 mutations may play a significant role in the efficacy and prognosis of venetoclax-based therapy in P53 mutant AML patients. Therefore, for P53 mutant patients with concurrent FAT1 mutations, it may be more reasonable to use venetoclax combination therapy, as this could potentially improve their prognosis.

## 4 Discussion

The role of FAT1 in tumors has attracted increasing interest from researchers, with FAT1 being considered an emerging biomarker for tumor diseases and a target for new therapies or monitoring. Garg et al. reported a FAT1 mutation rate of up to 10% in AML patients carrying the FLT3-ITD mutation, identified through targeted sequencing (8 out of 80 samples) (25). Clinical studies have also found that FAT1 mutations may be associated with poor outcomes and relapse in normal karyotype AML patients undergoing allogeneic hematopoietic cell transplantation (HCT), suggesting that FAT1 could be a pathogenic driver in AML (26). This underscores the significance of FAT1 mutations in AML. Therefore, in this study, we analyzed the characteristics of FAT1 mutations in AML. Firstly, we downloaded the simple somatic mutations (SSM) data and clinical data from the LAML-KR cohort in the ICGC database. We also retrospectively collected next-generation sequencing (NGS) data and clinical data from 108 primary AML patients who received initial induction therapy with a venetoclax combination regimen (referred to as the Venetoclax-AML cohort). The analysis showed that the FAT1 gene mutation rate in the LAML-KR cohort was approximately 15% (31/205), with a nonsynonymous mutation rate of about 6% (13/205). All nonsynonymous mutations were missense mutations, primarily occurring in the cadherin repeat regions, consistent with previously reported FAT1 mutation types in solid tumors (25, 27).

Notably, our study found that patients with FAT1 mutations in the LAML-KR cohort had a higher tumor mutation burden (TMB) compared to non-mutant patients. TMB is currently recognized as a predictive biomarker for cancer immunotherapy response. Some researchers have reported that, using multi-omics datasets from The Cancer Genome Atlas (TCGA) program and cancer cohort datasets treated with immune checkpoint blockade (ICB), they analyzed the correlation between somatic mutations and TMB across various cancers. The results showed that among the 32 TCGA cancer types, melanoma had the highest proportion of high TMB (≥10/Mb) at 49.4%. Mutations in 376 genes, including FAT1, were significantly associated with increased TMB in various cancers, and FAT1 mutation characteristics were associated with favorable responses to immunotherapy (28). In a study on the prognosis and immune function of FAT family genes in non-small cell lung cancer (NSCLC), researchers identified FAT1 mutation types including missense mutations, truncations, and amplifications across the entire gene. Compared to wild-type samples, FAT1 mutant samples had significantly higher TMB. In the NSCLC cohort treated with ICB, FAT1 mutations were significantly associated with better objective response, sustained clinical benefits, and longer progression-free survival (29). These findings in solid tumors, where FAT1 mutations are associated with increased TMB, are consistent with our results.

To further understand the characteristics of FAT1 mutations in AML, we analyzed the clinical and molecular genetic features of FAT1 mutant patients in both the LAML-KR cohort and the Venetoclax-AML cohort. Limited by the completeness of patient data in the LAML-KR cohort database, 83 patients were included for further analysis. Among these patients, compared to wild-type patients, FAT1 mutant patients had higher mutation rates of P53, DNMT3A, FLT3, and NPM1 genes. In the Venetoclax-AML cohort, we only confirmed that the P53 mutation rate was higher in FAT1 mutant patients, while the mutation rates of DNMT3A, FLT3, and NPM1 genes showed no significant differences between FAT1 mutant and wild-type patients. This suggests a potential correlation between FAT1 mutations and P53 mutations. Therefore, we conducted further exploration in subsequent studies on the impact of FAT1 mutations on the efficacy and prognosis of AML.

In this study, we primarily investigated the impact of FAT1 mutations on the efficacy and prognosis of AML. The results showed that in the LAML-KR cohort, FAT1 mutant patients had a significantly higher complete remission (CR) rate after initial induction chemotherapy compared to wild-type patients, although this did not translate into a significant survival advantage. In the Venetoclax-AML cohort, FAT1 mutant patients had a higher overall response rate (ORR, defined as CR/CRi + PR) compared to wild-type patients. Additionally, FAT1 mutant patients showed a trend toward improved progression-free survival (PFS) compared to wild-type patients, although the difference in PFS was not statistically significant. These findings indicate that FAT1 mutations are associated with better chemotherapy efficacy, including in patients receiving venetoclax-based combination regimens, and may confer a survival benefit. This suggests that FAT1 mutations play a significant role in the chemosensitivity and prognosis of AML patients.

In fact, the role of FAT1 mutations in chemotherapy sensitivity and prognosis has already been demonstrated in solid tumors (30). A comprehensive proteomics and drug screening study in pan-cancer models confirmed that FAT1 mutations in head and neck squamous cell carcinoma (HNSCC) sensitize cells to the BET inhibitor JQ1, whereas esophageal squamous cell carcinoma (ESCC) and other cancers exhibit different resistance patterns (31). Additionally, studies have shown that FAT1 mutations are also associated with the efficacy of immunotherapeutic drugs (32). Current research on AML immunotherapy, such as bispecific T-cell engagers, CAR-T or NK cell therapies, and immune checkpoint inhibitors (ICIs), is rapidly advancing and may be most effective in patients with minimal disease burden (e.g., MRD-positive remission or early salvage therapy). Several recent clinical trials are evaluating these therapies in such patients (33, 34). The impact or predictive value of FAT1 mutations on the efficacy and prognosis of these AML immunotherapies is highly worthy of further investigation. Moreover, for newly diagnosed and relapsed or refractory AML patients who are not suitable for intensive chemotherapy and have not previously received venetoclax treatment, triplet therapies combining venetoclax, HMAs, and a third agent (immunotherapy drug or small molecule targeted inhibitor) are becoming increasingly common in clinical trial designs (35). Our results suggest that FAT1 mutations have significant clinical value in selecting appropriate venetoclax-based combination regimens and immunotherapies for AML patients.

In previous studies, FAT1 and P53 share functional similarities in solid tumors, particularly in promoting EMT. FAT1 regulates EMT through the MAPK/ERK signaling pathway; its knockdown decreases E-cadherin expression while increasing N-cadherin, vimentin, and Snail levels (36). Similarly, mutant P53 directly binds to the E-box of the proximal E-cadherin promoter region, suppressing its expression and thereby facilitating EMT (37). Surprisingly, our study found a certain association between FAT1 mutations and P53 mutations, suggesting that FAT1 may play an important role in P53 mutant AML. Therefore, we explored the impact of FAT1 mutations on P53 mutant AML patients. Due to limitations in the number of cases and completeness of clinical data, we only analyzed 14 patients with P53 mutations in the Venetoclax-AML cohort. The results showed that among P53 mutant patients, those with FAT1 mutations exhibited better initial induction chemotherapy efficacy and showed a trend toward improved PFS compared to wild-type patients. This suggests that the venetoclax combination regimen may partially improve the poor prognosis of P53 mutant AML patients with concurrent FAT1 mutations. Therefore, for P53 mutant patients with FAT1 mutations, it is recommended to consider using venetoclax combination regimens, as this may improve their treatment response and prognosis. It is important to note that the correlation between FAT1 mutations and lower FAT1 expression is controversial, and the biological function of FAT1 mutations and FAT1 expression in tumors is different. A study by Chinese researchers on the role of FAT1 in OSCC seems to support this notion (38). Similarly, a recent study by Su Il Kim et al. on the clinical significance of FAT1 gene mutations and mRNA expression in HNSCC also supports this point (39).

While our study is the first to report the potential clinical significance of FAT1 mutations in AML, several limitations must be acknowledged. First, the retrospective nature of the LAML-KR cohort analysis, compounded by incomplete clinical data, may affect the reliability of chemotherapy efficacy comparisons between FAT1-mutant and wild-type patients. Second, the mutation spectrum observed in this cohort differs from classical AML patterns and may reflect unique clinicodemographic features or selection biases. Third, in the Venetoclax-AML cohort, limited sample size constrained robust multivariable analyses, necessitating non-parametric methods. While trends in survival outcomes were observed, statistical power for detecting FAT1-specific effects was insufficient. These findings underscore the need for validation in larger, prospective cohorts with extended follow-up. Our findings indicate that FAT1 mutations are beneficial for AML patients, especially those with P53 mutations, in terms of response to venetoclax-based combination therapy and possibly improving prognosis. This has important clinical implications for treatment selection and prognosis assessment in AML patients, and supports individualized treatment approaches for AML. We believe that with increasing research on FAT1, there will be new insights and strategies for understanding the role of FAT1 in the development and progression of hematologic and solid tumors, as well as in overcoming tumor drug resistance.

## Data Availability

The original contributions presented in the study are included in the article/supplementary material. Further inquiries can be directed to the corresponding authors.
